# Toxicokinetics of benzotriazole UV stabilizer UV-P in humans after single oral administration

**DOI:** 10.1007/s00204-024-03907-y

**Published:** 2024-11-29

**Authors:** Corinna Fischer, Julia Hiller, Edgar Leibold, Thomas Göen

**Affiliations:** 1 Institute and Outpatient Clinic of Occupational, Social, and Environmental Medicine, Friedrich-Alexander-Universität Erlangen-Nürnberg, Henkestraße 9–11, 91054 Erlangen, Germany; 2 BASF SE, Carl-Bosch‑Straße 38, 67056 Ludwigshafen Am Rhein, Germany

**Keywords:** Benzotriazole ultraviolet stabilizer, UV-P, Human metabolism, Human biomonitoring, Toxicokinetics

## Abstract

UV-P (2-(2*H*-Benzotriazol-2-yl)-p-cresol) is used as an ultraviolet (UV) light absorber in coating products, paints, adhesives, and sealants. Due to its widespread industrial and consumer uses, human exposure to UV-P is conceivable. In the study presented herein, initial data on its human in vivo metabolism were obtained for three study participants after single oral administration of 0.3 mg of UV-P/kg body weight. Urine and blood samples of two volunteers were collected up to 48 h after exposure. The third study participant donated urine and blood samples up to 72 h. Maximum levels of UV-P in blood of 184 ± 36 µg/l (85 ± 3% as conjugates) were reached 2.4 ± 1.2 h post-exposure. Maximum excretion rates of UV-P in urine of 2896 ± 884 µg/h (completely conjugated) were reached 3.5 ± 1.1 h post-exposure. 37.2 ± 5.4% of the orally administered dose of UV-P was recovered in urine within 48 h post-exposure. The present study provides insight into the complex absorption, distribution, metabolism, and elimination (ADME) processes of benzotriazole UV stabilizers (BUVS). The study also demonstrates differences in the ADME between sterically hindered BUVS, such as UV-327 and UV-328, and sterically unhindered BUVS, such as UV-P, in which the phenolic hydroxyl group is readily accessible for conjugation with glucuronic acid or sulfate.

**Supplementary Information:**

The online version contains supplementary material available at 10.1007/s00204-024-03907-y.

## Introduction

2-(2*H*-Benzotriazol-2-yl)-p-cresol (UV-P, CAS 2440–22-4) is used as an ultraviolet (UV) light absorber in various materials such as polymers, plastics, adhesives, and elastomers (ECHA 2017). UV-P is registered under the REACH (registration, evaluation, authorization, and restriction of chemicals) regulation; 1000–10000 t of UV-P are manufactured in and/or imported into the European Economic Area every year (ECHA 2024). It has already been demonstrated that UV-P is widely distributed in the environment; the substance has, for example, been detected in sediments (Nakata et al. 2009; Vimalkumar et al. 2018), bodies of water (Vimalkumar et al. 2018; Liu et al. 2014; Montesdeoca-Esponda et al. 2012), and in marine animals (Vimalkumar et al. 2018; Kim et al. 2011; Peng et al. 2017). UV-P has additionally been found in several clothing textiles, which were bought in clothing stores in Stockholm, Sweden (Avagyan et al. 2015) as well as in beverage bottle caps purchased from supermarkets in Tokyo, Japan (Sakuragi et al. 2021). As a result, human exposure to UV-P may occur via the consumption of fish and seafood or through the use of consumer products containing UV-P. Inhalation of indoor air may represent another potential route of exposure as UV-P was detected in indoor dust (Carpinteiro et al. 2010). Human exposure has already been demonstrated as UV-P has been detected in breast milk (Kim et al. 2019; Lee et al. 2015; Sun et al. 2022) and urine samples. With a mean concentration of 1.6 µg/g creatinine, UV-P was found to be the most predominant benzotriazole ultraviolet stabilizer (BUVS) in urine samples obtained from 182 adults living in Quzhou, China (Mao et al. 2024).

Despite the ubiquitous use, environmental distribution, and potential human exposure to UV-P, little is known about its toxicity. There are conflicting results published from in vitro studies regarding potential anti-androgenic or estrogenic activity. However, in vivo studies did so far give no indication of endocrine effects of UV-P (Fent et al. 2014; Zhuang et al. 2017; Sakuragi et al. 2021). UV-P activates the aryl hydrocarbon receptor (Fent et al. 2014; Kubota et al. 2022; Liang et al. 2023; Nagayoshi et al. 2015) and was found to bind to human serum albumin, which may disrupt its biological functions (Zhuang et al. 2016). Moreover, the substance was found to cause allergic contact dermatitis (Arisu et al. 1992; Björkner and Niklasson 1997; Niklasson and Björkner 1989).

UV-P was included in the human-biomonitoring (HBM) initiative agreed upon in 2010 by the German Federal Ministry for the Environment, Nature Conservation, and Nuclear Safety and the German Chemical Industry Association with the goal of developing appropriate human-biomonitoring strategies to assess potential exposure of the general population (Kolossa-Gehring et al. 2017). To receive an understanding of the absorption, distribution, metabolism, and elimination (ADME) processes of UV-P, and for the validation of a proper HBM parameter, the toxicokinetics of UV-P after oral administration has been explored by a human volunteer study.

## Materials and methods

### Chemicals and reagents

UV-P, *N,O*-bis(trimethylsilyl)acetamide in combination with 5% trimethylchlorosilane (BSA/TMCS), 1-(trimethylsilyl)imidazole (TSIM), acetonitrile (GC grade), ammonium acetate, chloroform (GC grade), glacial acetic acid, isopropanol (GC grade), sodium hydroxide (NaOH), and toluene (GC grade) were purchased from Merck KGaA (Darmstadt, Germany). 2-(2-Hydroxy-5-methylphenyl)benzotriazole-d_4_ (d_4_-UV-P) was ordered from ASCA GmbH Angewandte Synthesechemie Adlershof (Berlin, Germany). *β*-Glucuronidase/arylsulfatase from *Helix pomatia (H. pomatia)* was ordered as solution (10.8 U/ml of *β*-glucuronidase and 25 U/ml of arylsulfatase) from Roche Diagnostics GmbH (Mannheim, Germany). Double-distilled water was prepared using a Milli-Q system (Millipore, Bedford, USA). Human blood was donated by a volunteer participating in the in vivo study.

### Study design

Three healthy volunteers (two men and one woman) aged between 23 and 57 years were included in the study. Table 1 summarizes further information on the study participants. The administered dose of UV-P was calculated with respect to an adequate distance from the no-observed-adverse-effect level (NOAEL) determined in toxicological studies in rats (30 mg/kg body weight per day) (OECD 2017). The NOAEL was used as a starting point and a safety factor of 100 was applied. As a result, 0.3 mg/kg body weight was administered to each participant.

**Table 1:** Characteristics of the participants receiving a single oral dose of UV-P (0.3 mg/kg body weight)

Subject	Gender	Age [years]	Body weight [kg]	Duration of sample collection [h]	Number of urine samples	Number of blood samples
1	male	57	82	72	35	14
2	male	26	90	48	25	10
3	female	23	45	48	26	10

One blood and one urine sample were collected from each study participant prior to the oral administration of UV-P to determine potential background exposure. 14–27 mg of UV-P (see Table 1) were weighed onto a small piece of bread with butter, which was then consumed by the volunteers. From Subject 1, urine and blood samples were collected up to 72 h post-application. From the other two subjects, samples of urine and blood were collected up to 48 h post-application. The participants collected all urine voids in separate containers. Sampling times and volumes were recorded for each sample. The urine samples were aliquoted and stored at − 20 °C until analysis. 9 ml of blood were drawn from peripheral veins 1 h, 2 h, 3 h, 4 h, 5 h, 6 h, 10 h, 24 h, and 48 h after exposure and collected in EDTA-Monovettes®. From Subject 1, additional blood samples were drawn 8 h, 12 h, 32 h, and 72 h after exposure. Blood samples were stored at – 20 °C until analysis.

The ethics committee of the Friedrich-Alexander-Universität Erlangen-Nürnberg approved the study protocol (22-329-B). All participants were informed about the goals and risks of the study and gave written, informed consent prior to inclusion. General inclusion criteria for study participation were an age of between 18 and 60 years, non-smoker status, and an absence of occupational exposure to UV-P. All participants met these criteria.

### Calibration procedure

Stock solutions of UV-P and d_4_-UV-P (1000 mg/l) were prepared in acetonitrile and stored in amber glass vials at 4 °C. Calibration standards were prepared by spiking human urine and blood with various volumes of UV-P spiking solutions, which were prepared by the appropriate dilution of the stock solutions (see Sects. “Analytical procedure for blood samples” and “Analytical procedure for urine samples”). The calibration curves were obtained by plotting the quotients of the peak area ratios of the analyte and internal standard against the spiked concentrations of the calibration standards.

### Analytical procedure for blood samples

The preparation and processing of the blood samples was based on a previously published method (Fischer and Göen 2023). The samples were stored at – 20 °C until analysis. The samples were thawed at room temperature and mixed on a roller mixer. 1 ml of each sample was pipetted into a separate 6-ml glass vial. 20 µl of the d_4_-UV-P spiking solution [1000 µg/l of d_4_-UV-P in a mixture of 0.9% NaCl solution and acetonitrile (v/v, 9:1)] was added. 1 ml of acetonitrile was added to the samples, which were then vortex-mixed for 1 min and centrifuged at 4000 × *g* for 15 min at room temperature. The supernatants were transferred to clean 6-ml glass vials. 0.5 ml of sodium acetate buffer (0.4 M, pH 5.0) and 10 µl of *β*-glucuronidase/arylsulfatase from *H. pomatia* were added. After brief vortex-mixing, the samples were incubated at 37 °C for 16 h. After incubation, the samples were transferred to conical-bottomed centrifuge tubes and diluted with 2.5 ml of water. 1 ml of acetonitrile and 700 µl of chloroform were added and the samples were vortex-mixed for 30 s and centrifuged at 2500 × *g* for 15 min. The lower organic phases were pipetted into separate 1.8-ml glass vials and evaporated to dryness, followed by the addition of 40 µl of toluene and 40 µl of BSA/TMCS. The samples were vortex-mixed and derivatized at room temperature for 10 min. Afterwards, 20 µl of 5% (v/v) TSIM were added. The samples were vortex-mixed and transferred into the micro-inserts of 1.8-ml glass vials. Finally, the samples were heated to 40 °C for 1 h and analyzed by GC–MS/MS.

A seven-point calibration curve in the range of 0.5–150 µg/l was applied. Calibration standards were prepared by spiking human blood with various volumes of the UV-P spiking solutions containing 1000 µg/l or 100 µg/l of UV-P in a mixture of 0.9% NaCl solution and acetonitrile (v/v, 9:1). One sample each of Q_low_ (low-concentration quality-control material; 1 µg/l) and Q_high_ (high-concentration quality-control material; 30 µg/l) was processed analogously to the samples in each analytical run. A reagent-blank sample, containing water instead of blood, was included in each analytical run as well. Any blank values were subtracted from the analytical results. The reliability criteria of the method are given in the Supplementary Information.

To quantify the unconjugated form of UV-P, the blood samples of all study participants were additionally processed without the addition of *β*-glucuronidase/arylsulfatase from *H. pomatia* and incubation at 37 °C for 16 h.

### Analytical procedure for urine samples

The preparation and processing of urine samples was based on a method published by Fischer and Göen (2021). However, a few modifications were made. The samples were thawed at room temperature, thoroughly mixed, and subsequently centrifuged at 1600 × *g* for 2 min. 200 µl of the supernatant were transferred into a 10-ml glass, conical-bottomed centrifuge tube. 800 µl of water, 10 µl of the internal-standard spiking solution (10 mg/l of d_4_-UV-P in acetonitrile), and 1 ml of sodium acetate buffer (0.4 M, pH 5.0) were added to the samples. After vortex-mixing, 10 µl of *β*-glucuronidase/arylsulfatase from *H. pomatia* were added and the samples were briefly vortex-mixed. Enzymatic hydrolysis was performed at 37 °C for 16 h. Afterwards, 2 ml of water, 700 µl of isopropanol, and 400 µl of chloroform were added. The samples were vortex-mixed for 30 s and centrifuged at 2300 × *g* for 15 min. The lower organic phases were pipetted into 1.8-ml glass vials and evaporated to dryness, followed by the addition of 40 µl of toluene and 40 µl of BSA/TMCS. The samples were vortex-mixed and derivatized at room temperature for 10 min. Afterwards, 20 µl of 5% (v/v) TSIM were added. The samples were vortex-mixed and transferred into the micro-inserts of 1.8-ml glass vials. Finally, the samples were heated to 40 °C for 1 h and analyzed by GC–MS/MS.

A nine-point calibration curve in the range of 10–5000 µg/l was applied for the analysis of the in vivo study samples. For calibration, two analyte spiking solutions containing 50 mg/l and 2 mg/l of UV-P were prepared in acetonitrile. One sample each of Q_low_ (20 µg/l), Q_med_ (medium-concentration quality-control material (500 µg/l), and Q_high_ (1500 µg/l) was processed analogously to the samples in each analytical run. A reagent-blank sample, containing water instead of urine, was included in each analytical run. Any blank values were subtracted from the analytical results. The reliability criteria of the method are given in the Supplementary Information.

The urinary creatinine content of the study samples was determined photometrically by the Jaffé’s method (Larsen 1972). To quantify the unconjugated form of UV-P, the urine samples of one study participant were additionally processed without the addition of *β*-glucuronidase/arylsulfatase from *H. pomatia* and incubation at 37 °C.

### GC–MS/MS analysis

A TRACE 1310 gas-chromatographic system, equipped with a TriPlus RSH autosampler and a split/splitless injector, was coupled to a TSQ 9000 triple–quadrupole mass spectrometer equipped with an advanced electron ionization (AEI) source (Thermo Fisher Scientific Inc., Waltham, USA). The temperatures of the transfer line and the AEI source were set at 280 °C and 250 °C, respectively. The temperature of the injector was set at 280 °C. 1 µl of each sample was injected in splitless mode with a purge flow of 3 ml/min to vent after 1 min. Chromatographic separation was performed on a (5% phenyl)-methylpolysiloxane low-bleed capillary column (HP-5 ms UI, 60 m × 250 µm × 0.25 µm, Agilent Technologies, Inc., Santa Clara, USA) at a constant flow rate of 1 ml/min using helium as a carrier gas. The initial column temperature was set at 80 °C (hold for 1 min). The temperature was subsequently increased to 125 °C at a rate of 30 °C /min (hold for 0.5 min), followed by an increase to 230 °C at a rate of 30 °C /min and, from this temperature, to 240 °C at a rate of 1 °C/min. Finally, the temperature was increased to 300 °C at a rate of 50 °C/min (hold for 5 min). Total analysis time was 23 min. The mass spectrometer was operated in Multiple Reaction Monitoring (MRM) mode. The mass transition with the highest intensity was selected as the quantifier ion. A second mass transition was used as the qualifier ion. UV-P and d_4_-UV-P were measured in timed acquisition mode. Table SI-1 gives the parameter-specific settings and retention times of UV-P and its internal standard.

### Data evaluation

Following an exploratory approach, we included all detectable results in the toxicokinetic analysis to elucidate human in vivo toxicokineetics as completely as possible.

Renal excretion rates (*R*_*E*_*,* in µg/h) of UV-P at a certain point in time were calculated by the following equation:

$$R_{E} = \frac{{c_{i} \times v_{i} }}{{t_{i} - t_{i - 1} }}$$

where *c*_*i*_ (in µg/l) is the concentration of UV-P in the urine sample *i*, *v*_*i*_ (in l) is the volume of the respective urine sample, *t*_*i*_ (in h) is the elapsed time value of sample collection, and *t*_*i–1*_ (in h) is the elapsed time value of the previous sample collection.

The renal excretion kinetics of UV-P was plotted as temporal progressions of the renal excretion rates *R*_*E*_ at the midpoint of the respective sampling periods (*t*_*i,m*_ in h), which were calculated as follows:

$$t_{i,m} = t_{{i{-}1}} + \frac{{t_{i} - t_{{i{-}1}} }}{2}$$

Excretion curves were prepared for each study participant by plotting the current excretion rates against the midpoints of the sampling periods (*t*_i,m_). The slope (*k*_*el*_, elimination-rate constant) of the ln-transformed excretion curves was used to calculate the elimination half-life (*t*_*1/2*_):

$$t_{1/2} = \frac{{{\text{ln}}\left( 2 \right)}}{{\left| {k_{el} } \right|}}$$

By summing the molar excreted amounts of UV-P, the cumulative excreted amount (in µmol) was calculated for each study participant:

$$\mathop \sum \limits_{i = 0}^{n} \frac{{c_{i} \times v_{i} }}{M}$$

where *M* (in µg/µmol) is the molar mass of UV-P.

Furthermore, urinary excretion factors (*F*_*UE*_), given as UV-P dose equivalents (percentages), were calculated to express the total excretion of UV-P in urine after 6 h, 12 h, 24 h, and 48 h:

$$F_{UE} = \frac{{CE_{i} }}{{M_{D} }} \times 100$$

where *CE*_*i*_ is the amount of UV-P excreted after 6 h, 12 h, 24 h, and 48 h (in µmol), and *M*_*D*_ is the ingested amount of UV-P (in µmol).

The concentration–time curves in blood were obtained by plotting blood levels against time. The elimination half-life (*t*_*1/2*_) was calculated as described above.

The share of conjugation of UV-P was determined from the relation of the urinary excretion rates/blood levels obtained with and without enzymatic hydrolysis. The slope of the linear fit describes the percentage of the unconjugated form of UV-P.

Microsoft Excel® was used for data processing and Origin® (OriginLab Corporation) was used for curve fitting.

## Results

**Fig. 1:**
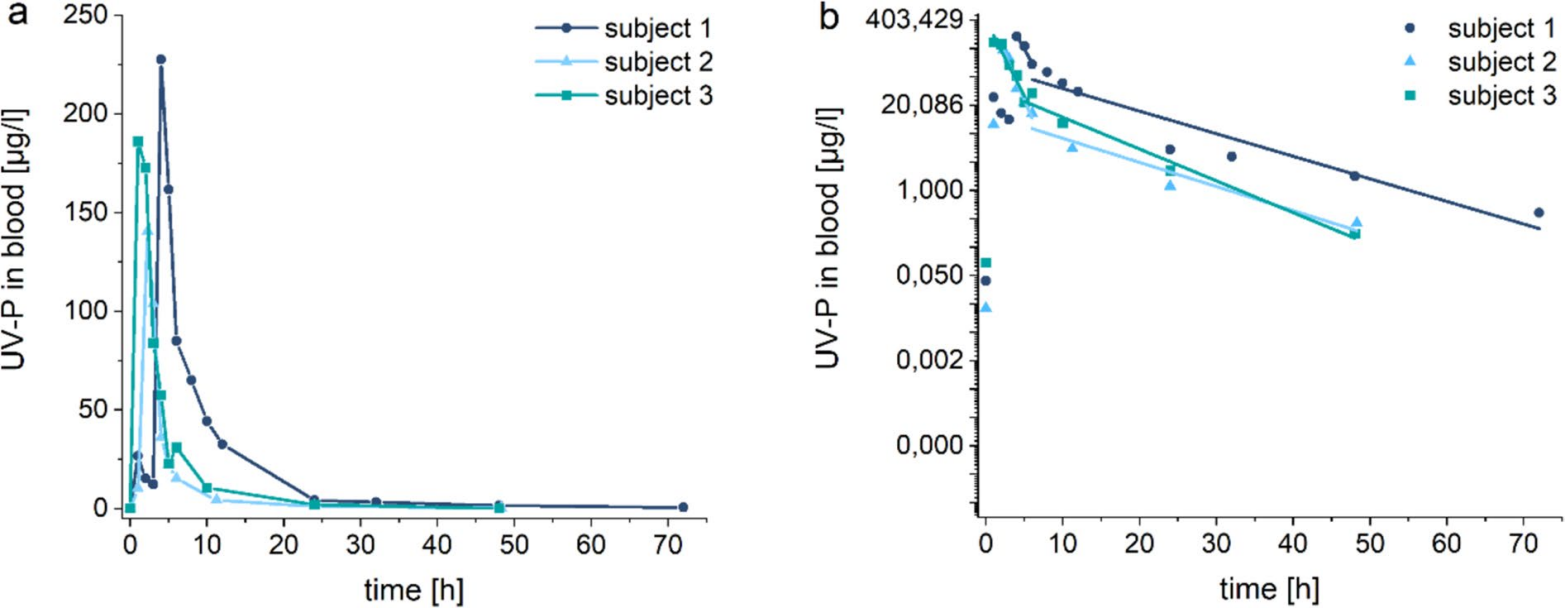
UV-P in blood (**a**) concentration–time curves of UV-P and (**b**) logarithmically scaled blood levels with linear fits

In the blood samples collected before oral administration, UV-P was not detected (< LOD). After exposure, UV-P was found in every blood sample of each subject. Figure 1a shows the concentration–time curves of UV-P in blood. The toxicokinetic data of UV-P in blood are summarized in Table 2.

**Table 2:** Kinetics of UV-P in blood after single oral administration [n = 3; mean ± SD (range)]

	UV-P
*c*_*max*_ [µg/l]	184 ± 36 (141–228)
*t*_*max*_ [h]	2.4 ± 1.2 (1.0–4.0)
*t*_*1/2*_—Phase 1 [h]	1.3 ± 0.1 (1.1–1.4)
*t*_*1/2*_—Phase 2 [h]	7.7 ± 1.1 (6.2–8.7)
Share of conjugation [%]	85 ± 3

The mean maximum blood levels of UV-P (184 ± 36 µg/l) were reached 1.0–4.0 h after oral administration. Afterwards, the concentrations decreased rapidly. Biphasic elimination kinetics were identified (see Fig. 1b). The first phase reached the maximum up to 6 h after oral administration and showed an elimination half-life of UV-P of 1.3 ± 0.1 h. Afterwards, the elimination rate decreased and an elimination half-life of 7.7 ± 1.1 h was determined. Six hour post-exposure, the mean blood levels of UV-P were 44 ± 30 µg/l, while 2.5 ± 1.3 µg/l of UV-P were detectable after 24 h. Forty-eight hours after exposure, 0.7 ± 0.7 µg/l of UV-P were measured. In the blood sample of Subject 1 collected 72 h after oral administration, 0.45 µg/l of UV-P were detected.

**Fig. 2:**
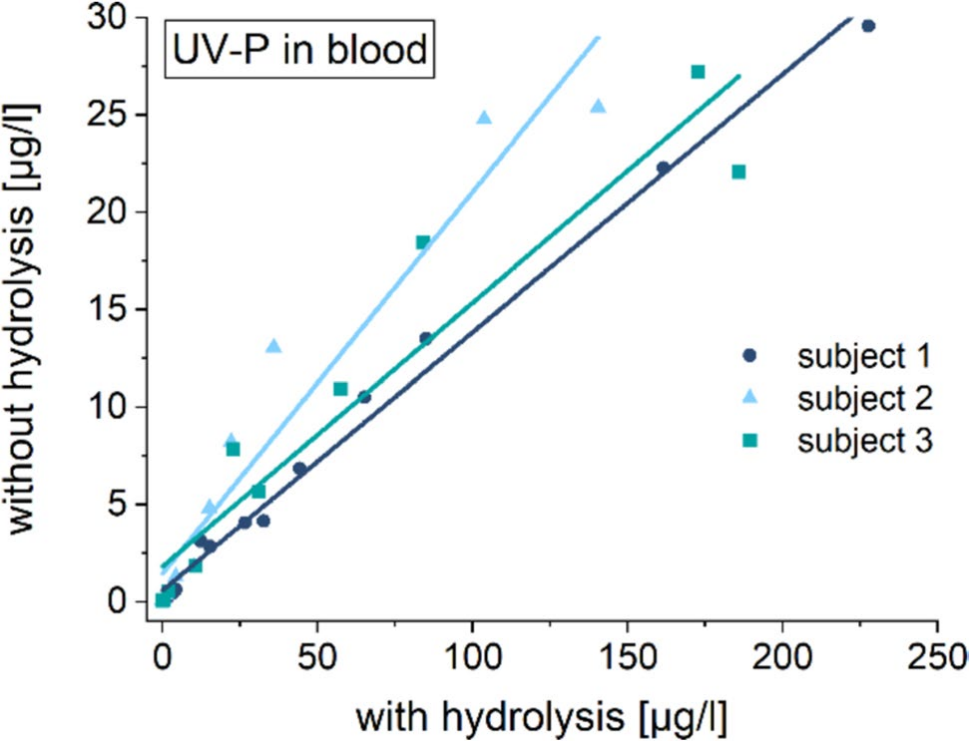
Blood levels of UV-P after processing with and without hydrolysis; lines: linear fits (slopes for subject 1: 0.133, subject 2: 0.196 and subject 3: 0.135)

Comparison of UV-P concentrations with and without enzymatic hydrolysis revealed that 85 ± 3% of the UV-P content in blood existed as conjugates (see Fig. 2).

**Fig. 3:**
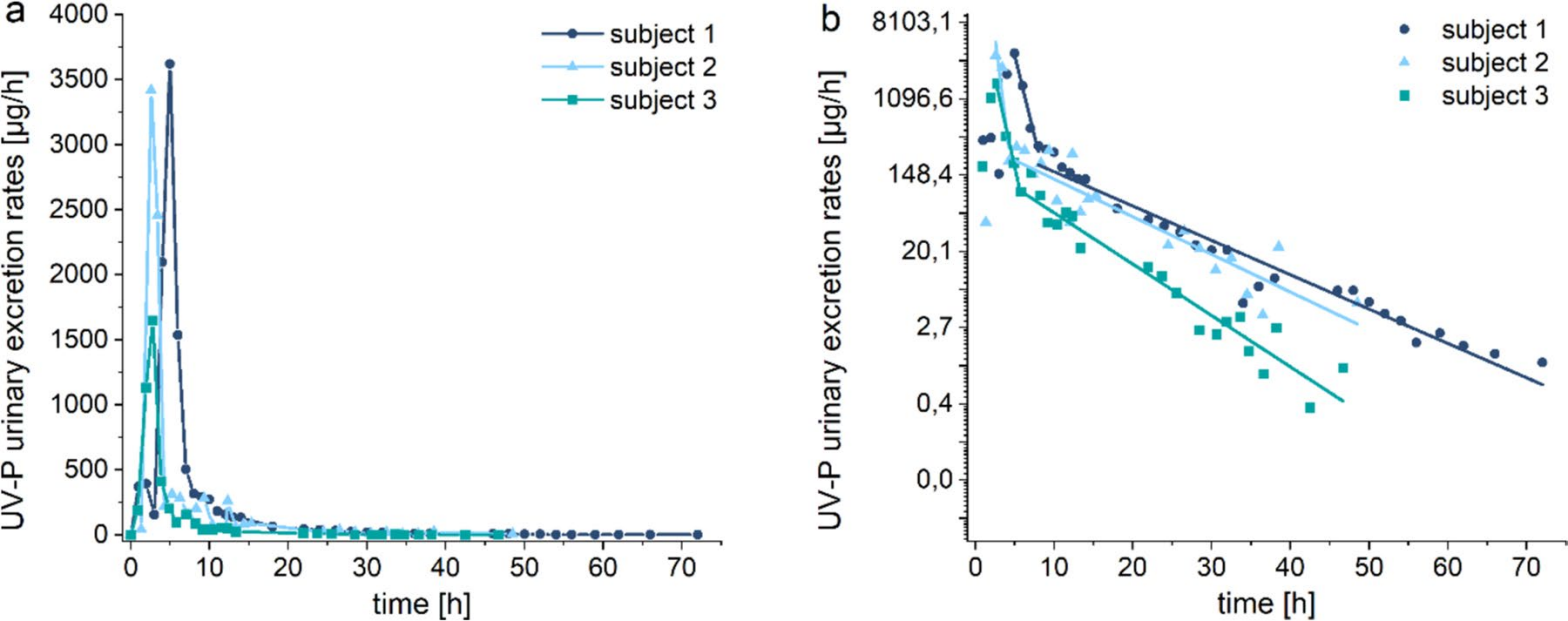
UV-P in urine (**a**) temporal progressions of the urinary excretions of UV-P and (**b**) logarithmically scaled urinary excretion rates with linear fits

In the urine samples collected before exposure, very low levels of UV-P were detected (0.9 ± 0.2 µg/l), whereas a strong increase in UV-P levels was found in the first urine void after oral administration (see Fig. 3a). The kinetics of UV-P in urine are summarized in Table 3. Maximum urinary excretion rates of 2896 ± 885 µg/h were reached after 2.6–5.0 h, followed by a brief, significant decline until 7 h post-administration with an elimination half-life of 0.7 ± 0.2 h (see Fig. 3b). The subsequent second elimination phase exhibited an elimination half-life of 6.6 ± 1.1 h. The cumulative urinary excreted amounts of UV-P are given in Figure SI-3. Within 6 h of exposure, 26.7–33.2% of the orally administered dose of UV-P was recovered in urine. While a total of 30.4–40.2% of the applied dose was recovered in urine within 12 h of exposure, 31.7–43.3% and 32.0–44.7% were recovered after 24 h and 48 h, respectively. For Subject 1, who donated urine for 72 h, 44.9% of the orally administered dose of UV-P was detected in the urine samples within 72 h. This subject also showed the highest recovery rates at earlier timepoints.

**Table 3:** Renal excretion kinetics of UV-P after single oral administration [*n* = 3; mean ± SD (range)]

	UV-P
*RE*_*max*_ [µg/h]	2896 ± 885 (1650–3621)
*t*_*max*_ [h]	3.5 ± 1.1 (2.6–5.0)
*t*_*1/2*_—Phase 1 [h]	0.7 ± 0.2 (0.4–0.8)
*t*_*1/2*_—Phase 2 [h]	6.6 ± 1.1 (5.1–7.7)
Cumulative amount excreted after 48 h [µmol]	35.6 ± 12.4 (19.0–48.9)
*F*_*UE*_ after 6 h [%]	29.5 ± 2.7 (26.7–33.2)
*F*_*UE*_ after 12 h [%]	34.2 ± 4.3 (30.4–40.2)
*F*_*UE*_ after 24 h [%]	36.3 ± 5.1 (31.7–43.3)
*F*_*UE*_ after 48 h [%]	37.2 ± 5.4 (32.0–44.7)
Share of conjugation [%]	99.9

*RE*_*max*_ = maximum renal excretion rate, *t*_*max*_ = timepoint of maximum renal excretion rate, *t*_*1/2*_ = elimination half-life, *F*_*UE*_ = urinary excretion factor

Processing the urine samples with and without enzymatic hydrolysis revealed that UV-P is almost entirely present in urine in its conjugated form (99.9%).

## Discussion

The kinetics of UV-P in blood exhibited rapid intestinal resorption of the compound. Maximum blood levels were reached 1–2 h post-exposure in two of the subjects and 4 h post-exposure in the third subject. The inter-individual variation may be explained by different dietary sequences as no specifications were given to the subjects regarding food intake. As a result, the volunteers ingested UV-P either on an empty stomach or after a meal. Since food intake delays gastric emptying and resorption, having a meal prior to UV-P application may explain the delayed *t*_*max*_ observed in Subject 1 compared to Subjects 2 and 3 (see Fig. 1a). Nevertheless, the resorption was distinctly faster than for the previously explored BUVS UV-327 and UV-328, for which maximum concentrations in blood were reached an average of 6 and 8 h, respectively, after oral administration (Fischer et al. 2023; Denghel et al. 2021). The comparatively fast and efficient resorption of UV-P may be explained by the lower molecular mass, lower lipophilicity (n-octanol/water partition), and higher water solubility compared to other BUVS, all of which promote resorption and inter-compartment penetration processes. This effect was recently demonstrated in comparative studies of the resorption and bioaccumulation of UV-P and other BUVS in zebrafish (Zhang et al. 2021) and juvenile rainbow trout (Leubner et al. 2023).

Blood and urine samples were collected from Subject 1 up to 72 h after exposure. The data of this subject demonstrated that, even 3 days after administration, traces of the compound were measurable in body fluids. Nevertheless, the cumulative amount excreted via urine reached a plateau about 24 h after exposure (see Figure SI-3), which showed that almost the total quantity of the compound was eliminated within this period. The data of the two other volunteers, who donated blood and urine samples up to 48 h after exposure, are consistent with this observation. The elimination half-lives were also comparable in all three volunteers. Compared to maximum blood levels, maximum urinary excretion rates were slightly delayed (2.6–5.0 h post-exposure). The maximum urinary excretion rate of Subject 3 was significantly lower compared to Subjects 1 and 2, which may be attributed to the significantly lower ingested dose of UV-P due to the significantly lower body weight of this subject. Nonetheless, the other kinetics data were similar for all three participants.

UV-P showed distinct differences to UV-327 and UV-328 regarding renal elimination kinetics. UV-P reached maximum urinary excretion rates after 3–5 h, whereas *t*_*max*_ of UV-327 and UV-328 was determined to be 13.5 h and 8.5 h, respectively (Denghel et al. 2021; Fischer et al. 2023). Moreover, UV-P was eliminated much faster than the other two BUVS with average half-lives of 0.7 h and 6.6 h for the two phases, compared to 18.2 h and 56.7 h for UV-327 and UV-328, respectively. As a crucial detail of the elimination process, UV-P was eliminated almost completely in its conjugated form, whereas the share of conjugation of renally eliminated UV-327 and UV-328 was found to be 16% each (Denghel et al. 2021; Fischer et al. 2023). The reason for the different efficacies of the Phase II metabolism of these substances may be the high accessibility of the phenolic hydroxyl group of UV-P for these reactions. In the other BUVS, the hydroxyl group is sterically hindered by proximate alkyl groups. Moreover, UV-P was excreted by a considerably larger extent via urine (32–45% of the applied dose during 48-h sampling), while only 0.003% and 0.001% of the ingested amounts of UV-327 and UV-328, respectively, accumulated until 72 h post-exposure. However, oxidative metabolites of UV-327 and UV-328 accounted for the majority of the renally eliminated amounts of these BUVS. Nevertheless, the sum of the renally eliminated parent compound and metabolites was only 0.12% (UV-328) and 0.03% (UV-327) (Denghel et al. 2021; Fischer et al. 2023).

In the study presented herein, possible Phase I metabolites of UV-P were not explored. However, the rapid and extensive conjugation and elimination suggest that metabolism may play a minor role in the toxicokinetics of UV-P. Moreover, a toxicokinetics study of unsubstituted 2-(2*H*-benzotrizol-2-yl) phenol and eight substituted phenolic benzotriazoles in Sprague–Dawley rats revealed oral bioavailability of 12.8–23% (Waidyanatha et al. 2021), which supports the assumption that the cumulated excretion amount of UV-P of 37% may represent the oral resorption of UV-P in humans.

The study possesses some strengths as well as limitations. A limitation of the study is the low number of participants. However, the recruited volunteers are made up of both sexes and cover a wide age-range. Moreover, the results show a proper conformity which do not indicate a high inter-individual variance. A major strength of the study is the double tracked approach with simultaneous monitoring of UV-P in blood and urine. Additionally, the consideration of phase II metabolism has been revealed as reasonable strategy.

## Conclusion

The human study presented herein provides the first information on the absorption, distribution, and elimination of the UV absorber UV-P in humans. The data obtained within this study indicate that UV-P is rapidly and extensively absorbed by the intestinal tract. Subsequently, the compound underwent an effective Phase II metabolism. The generated glucuronide and sulfate conjugates were eliminated quickly and efficiently via the urine. As such, the toxicokinetics of UV-P differ distinctly from those of BUVS such as UV-327 and UV-328, in which the substituent adjacent to the phenolic hydroxyl group may constitute a sterical hindrance for conjugation. The prompt renal elimination of UV-P, which was accelerated by a complete conjugation of the compound, may bypass potential Phase I metabolism. As a result, the cumulative renal elimination rate of 37% may represent the oral bioavailability of UV-P in humans. This effective renal elimination as well as the renal excretion factor provided by the present study supports the determination of UV-P in urine after enzymatic hydrolysis as an efficient and reliable HBM parameter for human exposure from the environment but also in occupational settings. This is clearly divergent from the HBM strategy for BUVS, whose renal elimination is inhibited due to sterical hindrance, where the determination of this compounds in blood or blood plasma is required.

## Supplementary Information

Below is the link to the electronic supplementary material.

Supplementary file1 (PDF 417 KB)

## Data Availability

Data are stored under controlled access at the Institute and Outpatient Clinic of Occupational, Social, and Environmental Medicine at Friedrich-Alexander-Universität Erlangen-Nürnberg. Anonymized raw data, not otherwise included in the article or online supplement, are available from the corresponding author upon reasonable request.
