# Supplementary material for: Toxicokinetics of benzotriazole UV stabilizer UV-P in humans after single oral administration
Source: Arch Toxicol. 2024 Nov 29;99(2):623–31. doi: 10.1007/s00204-024-03907-y (PMC11775033; doi:10.1007/s00204-024-03907-y)
Supplement: Supplementary file 1 — Supplementary file1 (PDF 417 KB) [file 204_2024_3907_MOESM1_ESM.pdf]

SUPPLEMENTARY INFORMATION

Human metabolism and excretion kinetics of benzotriazole UV stabilizer UV-P after single oral administration

Corinna Fischer[1], Julia Hiller[1], Edgar Leibold[2], Thomas Göen[1,*]

[1] Institute and Outpatient Clinic of Occupational, Social, and Environmental Medicine, Friedrich-Alexander-Universität Erlangen-Nürnberg, Henkestraße 9–11, 91054 Erlangen, Germany
[2] BASF SE, Carl-Bosch-Straße 38, 67056 Ludwigshafen am Rhein, Germany

[*]**Corresponding author:** thomas.goeen@fau.de

Table SI-1 Retention times and parameter-specific parameters of UV-P and d_4-UV-P

Analyte/ISTD	t_R [min]	Quantifier ion [*m/z*]		CE [V]	Qualifier ion [*m/z*]		CE [V]
		Precursor ion	Product ion		Precursor ion	Precursor ion	
d_4-UV-P	14.80	286.1	228.1	20	268.1	213.1	35
UV-P	14.83	282.1	224.0	20	282.1	209.0	35

ISTD = internal standard, t_R = retention time, *CE* = collision energy

Reliability of the determination of UV-P in blood

For the calculation of the limit of detection (LOD) and the limit of quantitation (LOQ), an equidistant ten-point calibration curve in the range of 0.2–2.0 µg/l of UV-P was prepared in human blood. The samples were processed and analyzed in triplicate according to the calibration-curve procedure (Bader et al. 2010; DIN 2008).

For validation, a nine-point calibration curve in the range of 10–2000 µg/l was applied. Accuracy was determined at three different concentrations (low-concentration quality-control material Q_{low}

(20 µg/l), medium-concentration quality-control material Q_{med} (200 µg/l), and high-concentration quality-control material Q_{high} (800 µg/l)) by calculation of the relative recovery rates of ten samples each. Precision and repeatability were determined by calculating intraday and interday relative standard deviations. To determine precision, samples spiked at three different concentrations (Q_{low}, Q_{med}, and Q_{high}) were processed and analyzed ten times in parallel. Repeatability was determined by processing and analyzing Q_{low} and Q_{high} samples on six different days. The validation data are summarized in Table SI-2.

Table SI-2 Limits of detection and quantitation, precision (n = 10), repeatability (n = 6), and accuracy (n = 10) for the determination of UV-P in blood

LOD [µg/l]	LOQ [µg/l]	Precision [%] (n = 10)			Repeatability [%] (n = 6)			Accuracy [%] (n = 10)		
		Q_{low}	Q_{med}	Q_{high}	Q_{low}	Q_{med}	Q_{high}	Q_{low}	Q_{med}	Q_{high}
0.05	0.17	1.6	1.6	2.8	4.1	4.4	2.7	94 ± 2 (90–95)	98 ± 2 (96–102)	102 ± 3 (99–107)

LOD limit of detection, *LOQ* limit of quantitation, Q_{low} low-concentration quality-control material (20 µg/l), Q_{med} medium-concentration quality-control material (200 µg/l), Q_{high} high-concentration quality-control material (800 µg/l)

The LOD and the LOQ were found to be 0.05 µg/l and 0.17 µg/l, respectively, indicating high sensitivity. The precision of the method was confirmed by relative standard deviations ranging from 1.6% to 2.8%. Repeatability was demonstrated by relative standard deviations of 2.7–4.4%. Furthermore, mean relative recovery rates in the range of 94–102% were achieved. The calibration curve was linear under the described analytical conditions. In summary, the method hereby presented enables the reproducible and accurate determination of UV-P in blood.

Lower UV-P levels are expected in the in vivo study samples. The calibration range was therefore adjusted, and a seven-point calibration curve in the range of 0.5–150 µg/l was applied to process and analyze the in vivo study samples. Figure SI-1 shows a representative calibration curve. No significant carryover effects were observed in any analytical series.

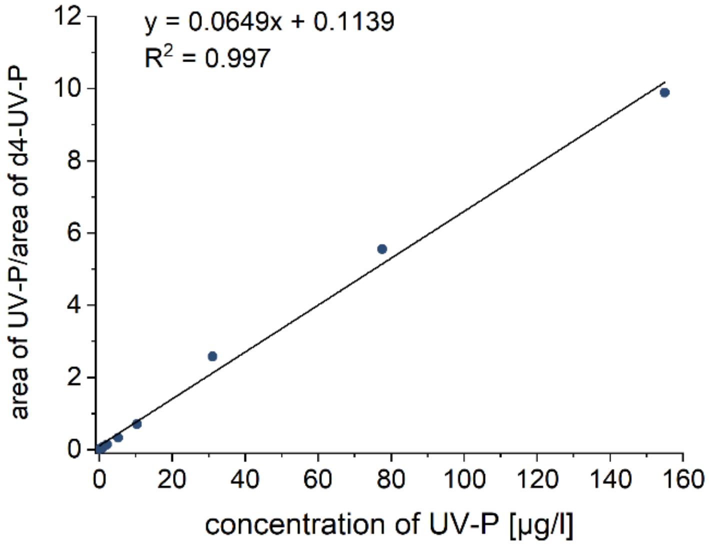

Fig. SI-1 Representative calibration curve for the determination of UV-P in blood

Reliability of the determination of UV-P in urine

For the calculation of the LOD and the LOQ, an equidistant ten-point calibration curve in the range of 0.2–2.0 µg/l of UV-P was prepared in human urine. The samples were processed and analyzed in triplicate according to the calibration-curve procedure (Bader et al. 2010; DIN 2008). Accuracy was determined at two different concentrations (low-concentration quality-control material Q_{low} (1 µg/l) and high-concentration quality-control material Q_{high} (20 µg/l)) by calculation of the relative recovery rates of six samples each. Accordingly, six individual urine samples with creatinine levels ranging from 0.4–2.2 g/l were spiked with UV-P, processed, and analyzed. The urine samples were additionally processed and analyzed without the addition of UV-P. The relative recovery rates were calculated based on the analyte concentrations in the spiked samples. Any background levels in the unspiked samples were subtracted from the analytical results. Precision and repeatability were determined by calculating intraday and interday relative standard deviations. To determine precision, samples spiked at two different concentrations (Q_{low} and Q_{high}) were processed and

analyzed six times in parallel. Repeatability was determined by processing and analyzing Q_{low} and Q_{high} samples on three different days.

The validation data are summarized in Table SI-3. The LOD and the LOQ were determined to be 0.06 µg/l and 0.21 µg/l, respectively, indicating high sensitivity. The precision of the method was confirmed by relative standard deviations ranging from 3.0% to 7.8%. Repeatability was demonstrated by relative standard deviations of 1.6–4.0%. Furthermore, mean relative recovery rates in the range of 91–99% were achieved.

Table SI-3 Limits of detection and quantitation, precision (n = 8), repeatability (n = 5), and accuracy (n = 8) for the determination of UV-P in urine

LOD [µg/l]	LOQ [µg/l]	Precision [%] (n = 6)		Repeatability [%] (n = 3)		Accuracy [%] (Mean (range), n = 6)	
		Q_{low}	Q_{high}	Q_{low}	Q_{high}	Q_{low}	Q_{high}
0.06	0.21	7.8	3.0	4.0	1.6	91 ± 3 (86–93)	99 ± 6 (90–107)

LOD limit of detection, *LOQ* limit of quantitation, Q_{low} low-concentration quality-control material (1 µg/l), Q_{high} high-concentration quality-control material (20 µg/l)

An extended calibration range with UV-P concentrations up to 5000 µg/l was applied to process and analyze the in vivo study samples. For the extended calibration range, repeatability was determined by processing and analyzing Q_{low} (20 µg/l), Q_{med} (500 µg/l), and Q_{high} (1500 µg/l) samples on four different days. Additionally, a urine sample with high levels of UV-P obtained from the in vivo study was prepared, processed, and analyzed in triplicate. Mean concentrations and relative standard deviations were subsequently calculated.

Relative standard deviations of 12.9% (Q_{low}), 8.5% (Q_{med}), and 5.7% (Q_{high}) were obtained. The mean concentration of UV-P in the sample with high levels of UV-P was found to be 4535 µg/l with a relative standard deviation of 1.4%. The calibration curve was linear under the described analytical conditions in the concentration range from 10 to 5000 µg/l. Figure SI-2 shows a

representative calibration curve. The method therefore allows an accurate determination of UV-P using the extended calibration range up to 5000 µg/l. No significant contamination as well as carryover effects were observed in any analytical series.

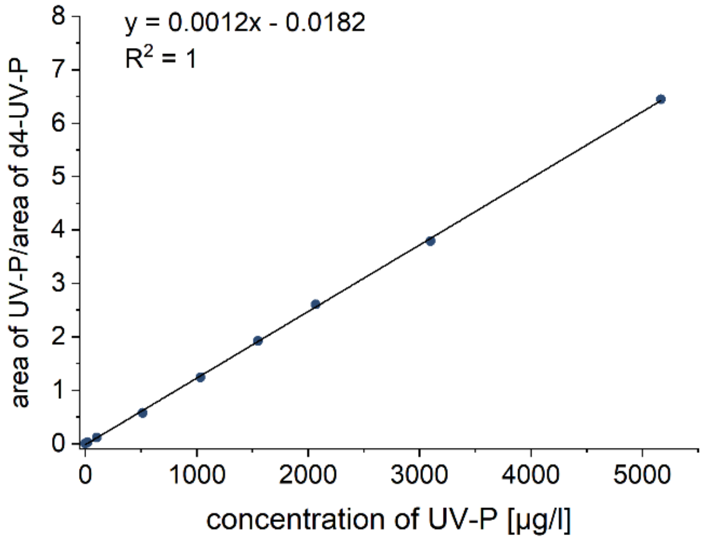

Fig. SI-2 Representative calibration curve for the determination of UV-P in urine

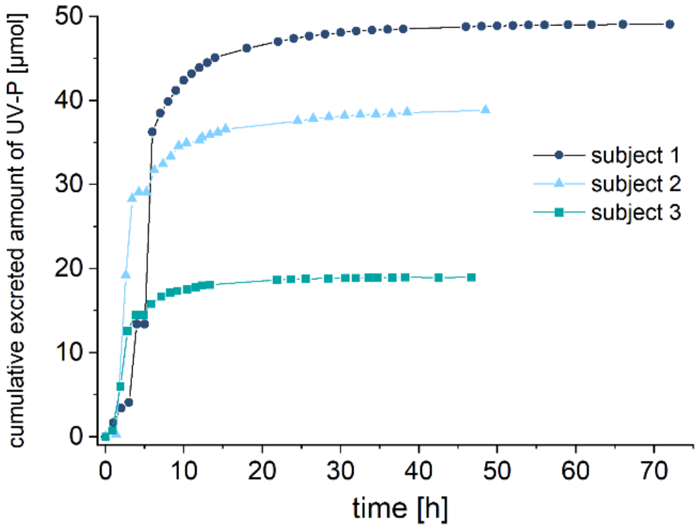

Fig. SI-3 Cumulative urinary excreted amounts of UV-P

SUPPLEMENTARY INFORMATION

References

Bader M, Barr D, Göen T, Schaller KH, Scherer G, Angerer J (2010) Reliability criteria for analytical methods. Biomonitoring methods. In: Angerer J., Hartwig A. (eds) The MAK-Collection for Occupational Health and Safety. Part IV: Biomonitoring Methods, vol 12. Wiley-VCH, Weinheim, pp 55–101

Deutsches Institut für Normung [German Institute for Standardization] (DIN) (2008) DIN 32645:2008-11: Chemical analysis - decision limit, detection limit and determination limit under repeatability conditions - terms, methods, evaluation
